# Promote Positive Behaviors in Preschoolers by Implementing an Innovative Educational Program for the Training and Development of Social and Emotional Skills (DeCo–S.E.)

**DOI:** 10.3390/ijerph192214931

**Published:** 2022-11-13

**Authors:** Adela Badau, Irina-Mihaela Trifan

**Affiliations:** Petru Maior Faculty of Sciences and Letters, George Emil Palade University of Medicine, Pharmacy, Sciences and Technology, 540142 Targu Mures, Romania

**Keywords:** preschoolers, DeCo–S.E., emotional regulation, positive behaviours, social-emotional skills, strategies, education

## Abstract

The purpose of our research is to determine emotional and behavioural modelling in an emotionally safe environment in a group of kindergarten preschoolers, following the application of an innovative curriculum project, designed and implemented for the first time, Educational Program for the training and development of social and emotional skills (DeCo-S.E.). Eighteen teachers, involved in the research, were divided into two samples: the experimental group (EG) consisting of 10 preschool teachers who were trained to take up the DeCo-S.E. program, and the control group (GC) including eight preschool teachers who applied the classic educational strategies. The DeCo–S.E. program is aimed at developing social and emotional skills (emotion identification, frustration tolerance), reducing behavior problems, and solving problems with peers as part of the training process. The study also included 142 children in their last year at kindergarten, aged X ± SD 5.87 ± 2.87 years old, divided into two groups: the EG consisting of 74 children to whom the experimental program was implemented and the GC comprising 72 children who did not take part in the training program. In the present study, we applied only the Preschool and Kindergarten Behavior Scales for Teachers (PKBS-2) questionnaire to children in the pre- and post-experimental phases. The results were processed with the statistical software SPSS 22. The analysis of the scores of the questionnaire applied to the children highlighted a significant improvement in EG on both scales. The study reveals the effectiveness of the Development of social and emotional skills programs in preschool children has proven its effectiveness by reducing undesirable/maladaptive behaviours and positively developing socio-emotional skills in preschool children.

## 1. Introduction

The formation of social and emotional skills starting from early childhood conditions the child’s long-term success, both personally and professionally [1,2]. Therefore, everything that happens in kindergarten has a significant impact on the child’s educational, mental, behavioural and social development [3,4,5]. The role of the teachers is very important in modelling children’s behaviours, as an expression of the particular way in which they express themselves. One of the most difficult aspects identified by teachers is the management of behavior problems that they have to manage in the classroom [6,7,8].

Our research dealt with aspects of early social-emotional education both from a theoretical and a practical perspective. The major objective of socio-emotional education is the formation of preschoolers’ social and emotional skills. The foreseen purpose of this type of early education consists in modelling desirable behaviours in children to successfully integrate them both in school and life. According to the educational system in Romania, preschool education takes place in kindergartens and includes children aged between 3–6 years who attend the small group (3–4 years), the middle group (4–5 years) and the large group (5–6 years). According to the ISCED 2011 classification, children aged between 3–6 years belong to the Pre-primary education level, which in Romania is equivalent to Preschool education [9].

The Romanian Curriculum for early education was designed in 2019 [10] and emphasizes the child’s holistic development; however, for the socio-emotional domain, it presents only four dimensions of development and 12 behaviours that the child must show at the end of the preschool period. The four dimensions of development that must be formed in the preschool period aim at: interactions with adults and children of similar ages, prosocial behaviours, accepting and respecting diversity, self-concept, self-control and emotional expressiveness. These four dimensions are reflected in the following behaviours: the ability to accept and express feelings, the ability to react to changes, emotional regulation, controlling one’s own impulses, belonging to a group, cooperating with others, expressing disagreement, the ability to adapt to the expectations of other members, etc. [11]. In the absence of theoretical-applicative recommendations, the teacher uses the Curriculum only to derive the necessity to form these behaviours, without having, most of the time, the certainty of the scientific and methodological validity of their approaches. For these reasons, educators feel the need to know specific ways and strategies they can apply in practice, beyond theorizing concepts related to the social and emotional education of children [12,13]. In terms of practices and concrete ways of application, we observe a national limitation in this area of interest. In this context, our research positively and creatively approaches new research directions, promoting a curricular proposal, validated in this study. According to the statistics produced by the Ministry of Education in the period 2020–2021, for the year 2019, the participation in preschool education of children aged 4–6 was 82.3% compared to 95.4% in the EU [14]. The educational environment represents the foundation of social and emotional education, offering educators a powerful resource to build relevant learning experiences in which children can find contexts of free expression [15,16].

According to previous studies [17,18,19], it is important to combine and apply training strategies and techniques and to shape social and emotional skills in an optimal educational environment within early social and emotional education programs. Preschoolers, regardless of their medium of origin, cannot learn how to adapt and mobilize to achieve school success to reduce inappropriate behaviours that can appear in their childhood or adolescence [20,21] without specialized intervention. By participating in early social and emotional development programs, children will be able to form and maintain friendships, protect their peers, and have a positive attitude toward learning and social experiences [22,23]. They will be accepted in their group of friends and be able to adapt easily as they are more confident and have a bigger potential for academic success than others [24,25,26]. If an emotionally and socially safe educational environment is provided in the kindergarten and early intervention programs are applied, the risk factors that cause poor school performance and certain undesirable behaviours will be reduced [24,27].

Emotional education is a process through which children become autonomous and independent, managing to solve the problems they face, learn to understand and adjust their emotions, maintain positive relationships with others and take responsible decisions [28,29,30,31]. To make progress in the formation and development of social skills, preschoolers must be allowed to practice controlling their own emotions in an environment where they feel comfortable [32]. Additionally, children can develop a positive attitude toward themselves while developing socially, cognitively, and emotionally [33,34] are essential in learning conflict resolution techniques and strategies, especially in adopting pro-social behaviours [35,36].

The number of children/teacher ratio, for preschool education, is on average 15 compared to the EU where the average is 13 children/teacher [14]. The participation of educators in continuous training programs, which integrate content related to the effective management of the group of children contributes to the improvement of the management skills of undesirable behaviours that may appear in the community [37,38]. In this sense, kindergartens can become environments that allow preschoolers to develop their emotional and social skills and support them in acquiring some methods of managing possible behavioural problems [25].

Addressing the same issue [39,40], emphasize the importance of the moments that preschoolers spend in kindergarten because they represent a natural way of social, emotional, and cognitive learning. Learning contexts must be exploited because they represent a concrete moment of learning when explanations are given spontaneously [41,42]. Educators must provide behavioural feedback and discuss each concrete social learning situation systematically to train appropriate behaviours. Current research highlights the need to apply educational programs for social and emotional development from an early age to prevent possible behavioural problems [43,44,45,46].

The novelty of our research consists of two interventions, namely: setting up a space called The Active Relaxation Zone, focused on securing educational, emotional and social behavioural manifestations; applying the Educational Program for the training and development of children’s social and emotional skills (DeCo-S.E.), using techniques and strategies for training social and emotional skills in preschoolers.

The purpose of our research was to determine the level of emotional and behavioural modelling in preschoolers at kindergarten, following the application of the innovative curriculum project called Educational Program for the training and development of social and emotional skills (DeCo-S.E.) in an emotionally secure environment.

## 2. Materials and Methods

### 2.1. Participants

The present study included a sample of 146 subjects, 52% girls and 48% boys, with a mean age of X ± SD 5.87 ± 2.87 years old, who comes from an urban environment. The sample was divided into two groups, namely: the EG consisting of 74 children, of which 35 girls and 39 boys; the (CG consisting of 72 children, of which 41 girls and 31 boys. The participation of these subjects in the study was carried out with the informal and written consent of the parents.

The inclusion criteria of the sample of children were: they do not come from disadvantaged backgrounds, both parents have a stable income, they are not registered with neuro-motor disabilities, they fit into the age category, they participate in the training program throughout the study period, they complete the whole assessment. We note that none of the children included in the sample group was categorized as children with special educational needs. The exclusion criteria were: non-participation in the program implemented throughout the targeted period, failure to perform the evaluation, failure to meet the age criterion, and refusal of parental consent to participate in the study.

There were 18 teachers involved in this study, 10 of whom participated in the implementation of the DeCo-S.E. program intended for the experiment group, and the other eight formed the CG. The EGe had to implement the proposed program and set up the relaxation area, as well as coordinate all the specific activities. The selection of educators was made according to the possibilities of achieving the aspects targeted in the study, their availability and a minimum of 7 years of experience.

The preschoolers, who are in a large group at kindergarten, come from three different state preschool institutions from Tg. Mureș. All three educational institutions have the same capacity to educate preschoolers and apply the same curricular approach, respecting the provisions of the National Curriculum.

To determine the sample size, we performed a power analysis using G*Power. The results obtained regarding the calculated power is 0.80, which means that the minimum sample is 128, (64 subjects in each group). Thus, the condition regarding the size of the number of subjects per group to be able to make inter- and intra-group comparisons was respected.

### 2.2. Instruments

In the present research, we applied the Preschool and Kindergarten Behaviour Scale for Teachers-PKBS-2 questionnaire, which is a standardized instrument that evaluates the levels of social skills and problematic behaviours developed by preschoolers, in a variety of situations. The PKBS-2 [47], consists of a total of 76 items, divided into two scales, A and B, with a unique response to each item, according to a 4-point Likert type scale, as follows: never (1) rarely (2), sometimes (3) and often (4).

The Social Skills Scale—A includes three subscales:

▪ social cooperation (12 items): describes cooperative and self-regulating behaviours, which refer to those behavioural characteristics that are important when the preschooler has to follow the instructions received from adults;
▪ social interaction (11 items): refers to social initiation, to the behaviours that are important in initiating and maintaining friendships with others;
▪ social independence (11 items): reflects behaviours that are important in achieving independence and autonomy within the group of friends.

The Behaviour Problems Scale—B includes 42 items and is divided into two categories:

▪ externalizing problems (27 items): considers behavioural problems, such as aggression and antisocial behaviours.
▪ internalizing problems (15 items): refers to emotional problems superimposed on behavioural ones, such as social withdrawal, somatic problems and anxiety [47].

The questionnaire was translated in several stages. The first stage consisted of translation by specialists from the language centre within the university. In the second stage, the analysis and correlation with the specialized terminology of the translated version of the questionnaire were carried out by the committee of experts in education sciences. The validation of the questionnaire was carried out by the confirmatory analysis of the number of items/scales. Internal consistency validation is presented in Table 1.

We performed the confirmatory factor analysis to confirm whether the number and structure of the factors of each subscale are maintained after the translation and application of the questionnaire. In Table 2, we present the results of the confirmatory factor analysis, at a significance threshold of *p* < 0.01.

The high significance of the values of the two indexes justifies the implementation of the factorial reduction procedure. Scale A—the structure of the three subscales is preserved, the only items that are not relevant and were removed from the original version were: item 12—Unacceptably uses free time, item 21—Invites other children to play and item 26—Stands up for his/her rights. In Scale B—the structure of the five subscales is maintained, the only items that are not relevant and were excluded from the original version: item 3—Teases or makes fun of other children, item 34—Destroys things that belong to other children, item 40—Tells lies and item 42—Bothers and annoys other children.

The fact that certain items are not relevant is not an exceptional situation, the translation of the assessment instruments, such as questionnaires, frequently generates such circumstances. Thus, following the validation of the questionnaire, we consider that its application to our sample is confirmed.

### 2.3. Procedure

The research included three stages.

The pre-experimental stage was structured as follows: the selection of the kindergartens participating in the study, educators’ agreement to take part in the research and the training of the 18 educators, included in the study, during the summer holiday; the pre-experimental stage ended with the application of the questionnaire to the sample children group; the results were recorded as the initial testing phase. The phases of the pre-experimental stage:

▪ the educators who coordinated the EG participated in the Effective management of the group of children training course. The content of the training aimed at acquiring strategies for managing undesirable behaviours, techniques and methods for modelling pro-social behaviours, improving the interaction between educators and children, and ways to observe and evaluate children’s behaviours. The first author of this article trained the educators who were directly involved in the application of the educational project—duration 4 weeks, 80 h.
▪ the systematic observation of children’s behaviours and attitudes was carried out within the daily instructional and educational activities without the intervention plan proposed in this study—duration 6 weeks.
▪ the initial testing (IT) of the questionnaire was carried out by educators on the subjects included in the study– duration 2 weeks.

The experimental stage—with a duration of 14 weeks, in which the content of the DeCo–S.E. intervention program was applied only to the children included in the EG. The DeCo-S.E. program represents a flexible curricular offer with an integrated approach to the act of learning having the aim of training and developing the skills of interaction with adults and children of the same age, accepting and respecting diversity, to develop pro-social and relational behaviours in preschoolers.

The activities carried out targeted specific aspects of the proposed program: recognizing, understanding and managing emotions, emotional self-regulation, ways for preschoolers to establish positive interactions in the group room, modelling pro-social behaviours and assuming responsibility for their actions, according to Table 3.

The strategies used to implement the program focused on the following aspects:

▪ modelling desirable positive behaviours;
▪ using role play and dramatization;
▪ using routines and transitions;
▪ using, throughout the day, those moments that can represent a source of direct learning;
▪ using stories, rhymes, songs and puppets;

For theEG, simultaneously with the implemented educational program, the active relaxation area was set up in the group room to give children the space they need to practice emotional regulation throughout the daily program, with the active and attentive participation of the educators. This is an open area space, set up to provide visibility to the teacher to protect the children. The configuration of the space does not have to be rigid, but we recommend having a soft carpet, floor cushions/large fluffy pillows, a CD player with relaxing music, books, magazines, a mirror, the shiny bottle, boards through which children recognize emotions and learn steps for emotional regulation, anti-stress manipulative toys, an hourglass or clock, a basket of various stuffed toys, etc.

This area is intended to be used by all the children in the group at any time of the day when their emotions are too intense. Children will be explained how, when and how long they are allowed to use the active relaxation area. The time that preschoolers spend in this area can be determined by mutual agreement. They need to know that they are allowed to use this area when they feel upset, anxious, sad or angry. It is also essential that preschoolers feel safe in this area and be aware that the educator will be with them whenever they need it. To avoid using the active relaxation area too often just to avoid participating in other activities, a limited number of “relaxation tickets” have been provided, tickets that the child can use specifically in the morning and afternoon.

There was no educational intervention related to the research topic in theCG, and the educators who coordinated these groups not being involved in the training program.

**Figure 1:**
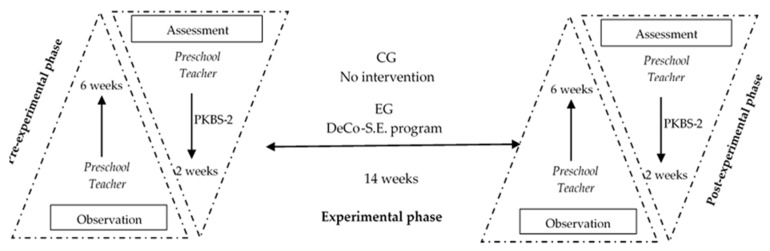
The diagram of the three stages of the research.

The post-experimental stage—with a duration of 8 weeks, in which both EG and CG samples had a final evaluation following the procedure from the pre-experimental stage, was completed with a new assessment by filling in the questionnaire for each child. For a more effective comprehension, we present a diagram of the three stages of the research we carried out, according to Figure 1.

### 2.4. Statistical Analysis

The resulting data were statistically processed with SPPS 22. To determine the sample volume, we performed the power analysis, using the G*Power program. To evaluate the reliability or internal consistency of the questionnaire, the statistical index Cronbach’s alpha (α) was calculated (Table 1). We also performed a confirmatory factor analysis to confirm if the number and composition of the items in the structure of each subscale are maintained after the translation and application of the questionnaire, we perform Bartlett’s Test for Sphericity, a significance level α = 0.01 and the Kaiser-Meyer-Olkin (KMO) value (Table 2).

The statistical analysis of the answers following the applied questionnaire aimed at the number of related points awarded by the subjects of the study by calculating the following indicators: arithmetic mean (X), standard deviation (SD), 95% confidence interval (CI) values were analysed to describe continuous variables such as a number of participants, the t-student test, for the significance threshold *p* < 0.05. In order to determine the homogeneity of the variants, we also statistically applied Levene’s Test for Equality of Variances, calculating the F indicator, at a *p* ≤ 0.005, and Cohen’s d effect size (d) (Table 4 and Table 5). The interpretation of Cohen’s d effect size was: 0.1–0.2 small, 0.3–0.5 medium, 0.5–0.8 large, and over 0.8 very large.

## 3. Results

We applied the comparative analysis on subscales to check if there are significant differences between the two samples, between tests, at a significance threshold of *p* < 0.05 (Table 4).

By analysing the recorded results, we find that the difference in the initial arithmetic averages was different; thus, for the social Cooperation subscale, it was 0.248, for social interaction 0.871, for social independence 0.330, and for social skills 0.797. In the final tests, statistically significant progress was registered in the EG compared to the CG one, taking into account the difference in arithmetic averages, thus in the social Cooperation subscale it was 2.16, in the social Interaction subscale 1.919, in the social Independence subscale 1.339, and in the social skills subscale, the total score was 5480.

According to Table 4, on the social Cooperation subscale, the EG registered statistically significant progress of 1959 among tests compared to the control one which registered a statistically insignificant progress of only 0.047, where *p* = 0.865; in the Social Interaction subscale, in theEG, the progress between the tests was statistically significant at 1.274, and in theCG, statistically insignificant at 0.226, where *p* = 0.430; in the Social Independence subscale, the progress recorded among test takers in the EG was statistically significant at 1806, and in the CG of statistically insignificant at 0.092, where *p* = 0.805; in the Social skills subscale, the progress recorded by the EGp was statistically significant at 5.102, while the CG progress is statistically insignificant at only 0.351, where *p* = 0.509. We consider that these significant differences were obtained by the EG as a result of the implementation of the DeCo-S.E. program.

To determine the homogeneity of the variants, we statistically applied Levene’s Test for Equality of Variances, at a *p*-value of ≤ 0.005, between the two groups in both tests. The obtained results between the EG and the CG were: for the Social Cooperation subscale at the initial testing, F = 0.117, *p* = 0.732 while at the final one F = 5.011, *p* = 0.027; at the Social Interaction subscale F = 4.300, *p* = 0.040, while at the final test F = 2.704, *p* = 0.102; in the Social Independence subscale F = 0.001, *p* = 0.977, in the final testing F = 0.222, *p* = 0.638; and in the Social skills subscale the total score was F= 1.134, *p* = *0*.289, while in the final test F = 3.617, *p* = 0.059. These results confirm the hypothesis of homogeneity of the variables, rejecting the null hypothesis. In EG, for all subscales, a medium effect size was recorded, the values falling between 0.395–0.498, and at CG for all subscales, the effect had a small effect, being below 0.200.

According to the statistical analysis of the results, it can be observed that there are no statistically significant differences between the two groups if we take into account the results of the initial tests; thus concerning the Self-Centred/Explosive subscale the difference in arithmetic means is 1.902, in the Attention problems/overactive subscale 1.115, in the Antisocial/ Aggressive 1.273, the Social Withdrawal subscale 0.356, the Anxiety/Somatic Problems subscale 0.160, and the total behavioural problems score is 3.776.

In the final tests, statistically significant progress was recorded in the EG compared to theCG, taking into account the difference in arithmetic averages; thus, in the Self-Centred/Explosive subscale the difference was 7520, in the Attention problems/overactive subscale 5311, in the Antisocial/Aggressive subscale of 4838, in the Social Withdrawal subscale 2239, in the Anxiety/Somatic Problems subscale 2523, and the total score of behaviour problems was 21,951. These results reveal the fact that in the EG behavioural problems were significantly reduced due to the intervention program and the introduction of the active relaxation area proposed in the study.

According to Table 5, on the Self-Centred/Explosive subscale, the EG registered a statistically significant progress of 5564 among tests compared to the CG which registered a statistically insignificant progress of only 0.047, where *p* = 0.757; on the Attention problems/overactive subscale, the EG registered a statistically significant progress of 3.935 compared to theCG, whose progress was insignificant by only 0.261; on the Antisocial/Aggressive subscale, the statistically significant progress of the EG was 3.935 and the CG recorded a statistically insignificant progress of 0.380; on the Social Withdrawal subscale, the EG registered a statistically significant progress of 2500, and the CG of 0.095 statistically insignificant; on the Anxiety/Somatic Problems subscale, the EG had a statistically significant progress of 2.532, and the CG only a statistically insignificant 0.135; in the subscale of Total score of behavior problems, the EG registered a statistically significant progress of 18,267, while the CG had a statistically insignificant progress of 0.107.

At Levene’s Test for Equality of Variance, at a *p*-value of ≤ 0.005 the results obtained by the two groups, per subscale between tests were: for the Self-Centred/Explosive subscale at the initial test F = 0.45, *p* = 0.704; at the final test F = 0.830, *p* = 0.364; on the Attention problems/overactive subscale at the initial test F= 0.122, *p* = 0.727, on the final test F = 12.322, where *p* = 0.001; in the Antisocial/Aggressive subscale at the initial test F = 0.576, *p* = 0.449, in the final test F= 13.172, where *p* = 0.000; in the Social Withdrawal subscale at the initial test F = 4.286, where *p* = 0.040, in the final test F = 0.233, with *p* = 0.630; for the Anxiety/Somatic Problem subscale, at the initial test F = 15.607, *p* = 0.000 and the final one F = 12.370, *p* = 0.001; for the Total score behaviour problems subscale at the initial test F = 0.494, *p* = 0.483, and the final test F = 15.695, *p* = 0.000; all these results reject the null hypothesis. In EG, for the Self-Centred/Explosive subscale, a large effect was recorded and in the other subscales, a medium effect, while in CG, in all subscales, the effect size was small, being below 0.200.

## 4. Discussion

### 4.1. General Framework

The present study aimed to determine the level of emotional and behavioural modelling in preschoolers in the large kindergarten group, following the application of the innovative curriculum project called Educational Program for the training and development of social and emotional skills (DeCo-S.E.) in an emotionally secure environment. The results of the study confirmed the effectiveness of the DeCo-S.E. training program in reducing behaviour problems by applying preschoolers’ emotional regulation strategies. Based on the analysis of the results of our study, following the implementation of the DeCo-S.E. program and the conception of the relaxation area, it can be observed that both on the scale of social skills and the scale of behaviour problems, the EG recorded significant progress compared to theCG. The results of our study contribute to expanding the level of knowledge and understanding of the impact that early socio-emotional education can have on preschoolers. The findings of our study complement previous research on this topic [48,49,50].

### 4.2. The Social Skills

Even if the initial level of social skills—scale A is in the area of high functionality, nevertheless its level increases under the influence of the innovative training program proposed and implemented by us, children acquire high social skills and the tendency to be extremely pleasant to their peers and adults advances even more. In the case of theCG, without the influence of the educational program, the situation remains unchanged, the differences between the initial and final test results, in this case, being statistically insignificant. The results of previous research confirmed the need for specialized intervention plans regarding the social skills of pre-schoolers [51,52]. The results and findings of our study regarding the need to implement innovative strategies adapted to the peculiarities and needs of pre-schoolers to acquire social skills are in accordance with recent studies, contributing in a practical way to offering an innovative and validated program with this program (DeCo-S.E.).

In line with our findings, we mention a study carried out on a Portuguese sample of 1030 children, of which 538 boys and 492 girls, aged between 3–6, where the Years Teacher Classroom Management (IY-TCM) training program was implemented and the entire PKBS questionnaire was applied; the study highlighted that on the social skills scale A, the number of children who shifted from the moderate or high- risk interval to the low-risk one was 8.7 times higher at the EG compared to those in the CG who moved in the opposite direction 6.0 times [53]. A study conducted using the same PKBS-2 instrument, on a sample of 300, taking into account the age of 5 years, recorded significant results, where *p* = 0.001 for all subscales of social skills, results that align with the results obtained in our study [54].

We mention another study conducted on a sample of 147 children aged 4–6 years old, who took part in a training program called the Aprender a convivir Program, implemented for a period of 12 weeks, which aimed to improve life and social skills; children who participated in this program recorded the following results: the EG to which the program was implemented to registered higher pre-test scores from a statistical point of view for scale A of social skills, where *p* = 0.001 [55]. A series of studies have addressed aspects related to the socio-emotional skills of preschoolers, demonstrating the importance of the impact of training programs in optimizing positive behaviours in pre-schoolers [56,57,58,59,60].

### 4.3. The Behaviour Problems

In the case of the assessed behavioural problems, Behaviors problems—scale B a decrease in their intensity is detected, statistically significant in theEG, because of the training program. Thus, in the case of the Egocentric/Temperament element, the significant reduction represents, according to the test manual, the transition from a moderate problem level to the average problem category. The results of our study regarding Behavioral problems—scale B are in line with the current guidelines and the findings identified by previous studies [17,61], contributing in this way to expanding the comprehension of the factors that influence behaviour and the ways to improve it in preschoolers.

If, before the implementation of the proposed innovative training program this fact meant the frequent engagement of children in antisocial behaviours, after completing the program the children rarely showed problems which might raise educators’ or parents’ concerns. In the case of attention problems/hyperactivity, the decrease determines the same type of reframing, from the moderate level to the borderline one, an improvement of the pro-sexual level and only isolated manifestations of hyperactivity are observed. These occasional manifestations seem to have been maintained only in the conditions of children’s unsatisfied desires, normal in the instructive educational process or at the end of the activities, in case of fatigue. In the case of the antisocial/aggressive component, the decrease in enrollment (from moderate to borderline) also means changes in children’s behaviour, with the antisocial and aggressive reactions being significantly reduced. In the case of the component related to social isolation, there is a reduction from a level considered high, with frequent manifestations of reticence, poor communication, and group avoidance, to a type of manifestation with moderate frequency, in children after the implementation of the training program becoming more open to social interactions, more communicative and open to other children and teachers. Regarding anxiety and somatic problems, the decrease in frequency is found within the same categories, moderate, from the maximum limit to the minimum one. The children presented fewer somatic complaints (“my stomach hurts” or “my head hurts”) and were less anxious, they approached new, unfamiliar situations with greater confidence.

The results presented in this study are confirmed by the findings of similar research conducted in Spain [17], Israel [62], and Chile [22], according to which the application of the curriculum project contributes to the reduction of undesirable behaviours and antisocial and aggressive reactions; moreover, children’s actions are shaped positively and they manage to regulate their emotions more easily.

In agreement with our study, other studies carried out on pre-schoolers from Portugal and Angola, have shown that behavioural problems are more likely to be externalized than repressed as a result of cultural influences [63]. A study performed in 2007 in Argentina [19] on a sample of 5697 children highlighted that almost 14.6% of children have internal behaviour problems while 14.7% demonstrate external behaviour ones, concluding that an early preventive approach to maladaptive behaviours is necessary. In line with our findings, we mention a study carried out on a Portuguese sample of 1030 children, of which 538 boys and 492 girls, aged between 3–6, where the Years Teacher Classroom Management (IY -TCM) training program was implemented and the entire PKBS questionnaire was applied; the study considering the behaviour problems—scale B, the recorded results showed that the number of children who shifted from the moderate or high-risk range to the low-risk one was 3.3 times lower during the experiment compared to the CG in which children moved in the opposite direction 1.6 times [53].

The study mentioned earlier confirms our results, concluding that if effective intervention is carried out through a training program on pre-schoolers’ socio-emotional and behavioural qualities, it has a positive and dynamic effect on pre-schoolers’ attitudes and skills. Another study carried out in Spain, on a sample of 147 children, aged between 4–6 years, who participated in a training program called the Aprender a convivir Program, implemented for three months, using the PKBS tool, registered at the EG where the program was implemented, statistically significant pre-test values in the reduction of problematic behaviors compared to the CG, where *p =* 0.001 [55]. A study carried out on a sample of 300 children between 3–6 years, using the same PKBS-2 test tool, found for children with an average age of 5 years, that the external behavioral aspects recorded an X ± SD = 1.293 ± 0.585, where *p* = 0.003, and internal behaviour problems X ± SD = 1.297 ± 0.568, where *p* = 0.001, results that align with the results identified in our study [54].

The relevance of the results of this study should be understood considering the formative and socio-emotional aspects of the first 5–6 years of children’s development regarding the impact of training and subsequent adaptation to school and the formation of successful relationships throughout life, conclusions supported by other studies [64,65,66,67,68].

### 4.4. The Active Relaxation Zone

In addition to the educational program applied to the EG, a significant contribution was made by the conception of the Active Relaxation Zone, the strategies and techniques used to model desirable behaviours, as well as through the training program that the educators went through, a fact confirmed by other studies as well [26,69]. Preschoolers’ development in a safe environment that offers them the possibility to acquire positive thinking is highlighted in pro-social behavioural manifestations, according to previous studies [70,71,72,73], and represents the most important predictor of human progress on all levels.

The creation of physical and social learning environments in kindergartens favours the adoption of holistic approaches that can influence responsiveness to children, intentional teaching, planning and implementation as well as evaluation and monitoring [74,75,76,77]. A study carried out by Blewitt C. et al. identified what are the main barriers to the education of preschoolers from the perspective of the educators, mentioning the safety of the educational environment that aims at the safety of exploring the world and social interactions [78]. In this context, the organization of spaces that provide safety for children positively influences the educational process, an idea also supported by Ng, S.C. et al. [79]. O’Conner et al., consider that in addition to the application of a curriculum aimed at the socio-emotional development of preschoolers, the modification of physical space to support positive emotions, represents an additional strategy for the development of socio-emotional behaviors [34]. The previously mentioned studies confirm the intervention in our study, setting up an active relaxation area, considering that it is an important factor in ensuring an optimal educational environment.

Among the limitations of the research, we mention: the sample of participants was a relatively small one, the design of the innovative program was based on the national educational and cultural background; the research undertaken was based on the results obtained in the first year of implementation of the intervention program; the relatively short duration of the intervention program; the documentation of how the curriculum was faithfully implemented was based only on oral reports from meetings held with the educators during the course of the experiment.

## 5. Conclusions

According to our results, we believe that the intervention program contributed to the formation of social and emotional skills that facilitate the modelling of pro-social behaviours, thus reducing the presence of undesirable behaviours in pre-schoolers.

Enriching educators’ knowledge by participating in training courses that highlight strategies and techniques for training and developing socio-emotional behaviours in pre-schoolers is extremely important. Thus, educators will be able to offer support to children in need, especially in the early education stage.

The results showed that during the research period the level of conflicts decreased significantly in the groups where the innovative curriculum project proposed by us (DeCo-S.E.) was applied, while in the CG the level of conflicts increased. The increased values of the level of closeness obtained after the implementation of the innovative training program indicate a closer and warmer relationship, with communication between the educators and the pre-schoolers from the EG being more effective.

By participating in the activities included in the curriculum project, the pre-schoolers in the EG were allowed to practice controlling their emotions in an environment where they feel comfortable by setting up an active relaxation area, a fact that generated a significant increase in the levels of social and emotional skills and a reduction in behavioural problems.

## Figures and Tables

**Table 1:** Reliability analysis for scale A and scale B of the PKBS 2 questionnaire.

Preschool and Kindergarten Behaviour Scale (PKBS-2)	Initial Testing		Final Testing	
	α-Cronbach’s	Items	α-Cronbach’s	Items
Scale A				
Social cooperation	0.890	12	0.886	11
Social interaction	0.880	11	0.879	10
Social independence	0.763	11	0.780	10
Scale B				
Self-Centred/Explosive	0.926	11	0.915	11
Attention problems/Overactive	0.914	8	0.901	8
Antisocial/Aggressive	0.819	7	0.854	3
Social Withdrawal	0.933	8	0.916	8
Anxiety/Somatic	0.846	8	0.931	8

**Table 2:** Values of KMO and Bartlett’s Test per subscale, PKBS 2.

KMO and Bartlett’s Test		Initial Testing		Final Testing	
		Scale A	Scale B	Scale A	Scale B
Adequacy		0.832	0.928	0.841	0.927
Bartlett’s Test of Sphericity		2701.203	5205.460	2702.144	5198.568
	df	561	861	559	860
	Sig.	0.000	0.000	0.000	0.000

df—degree of freedom, Sig.—statistical significance.

**Table 3:** Content of the DeCo-S.E. program.

Educational Activities DeCo–S.E.			Targeted Competencies			
	Development of Self-Concept	Developing Emotional Self-Control	Development of Emotional Expressiveness	Accepting and Respecting Diversity	Communication, Relationships and Interactions with Others	The Development of Pro-Social Behaviour
	Emotion management activities					
Expressing your own emotions and feelings	X	X	X		X	
Recognizing other people’s emotions	X	X	X	X		X
Emotion management and regulation	X	X	X	X	X	X
	Activities to learn social skills					
Establishing positive interactions with children		X		X	X	X
Collaboration and teamwork		X		X	X	
	Behavioural Problem-Solving Activities					
Expressing one’s feelings constructively	X	X	X	X	X	X
Solving problems in interaction with children	X	X		X	X	X
Demonstrating empathy toward others	X	X	X		X	X
	Activities to learn desirable behaviours in the group room					
Solving the problems arising in the interaction with children	X	X	X	X	X	X
Perception of rules and their effects	X	X		X	X	X

X—competencies targeted in the program.

**Table 4:** The centralizer of results according to social skills—Scale A.

Subscale A	Group	Testing	X	DX (TF-TI) ± SD	CI95%		t	*p*	d
					Lower	Upper			
Social cooperation	EG	TI	41,772	1.959 ± 4.943	3.207	0.696	3.108	0.003	0.461
		TF	43,731						
	CG	TI	41,524	0.047 ± 0.278	0.601	−0.505	0.171	0.865	0.173
		TF	41,571						
Social interaction	EG	TI	36,000	1.274 ± 4.439	2.401	0.146	2.260	0.027	0.412
		TF	37,271						
	CG	TI	35,129	0.226 ± 0.285	0.793	−0.340	0.793	0.430	0.157
		TF	35,352						
Social independence	EG	TI	38,000	1.806 ± 3.788	2.768	0.844	3.754	0.000	0.395
		TF	39,811						
	CG	TI	38,330	0.092 ± 0.335	0.751	−0.584	0.248	0.805	0.146
		TF	38,422						
Total score	EG	TI	115,779	5.102 ± 6.205	6.067	1.900	3.823	0.000	0.498
		TF	120,811						
	CG	TI	114,982	0.351 ± 0.468	10.729	−6.586	1.087	0.509	0.107
		TF	115,331						

X—arithmetic mean; DX—the difference between testers; SD—standard deviation; CI95%—95% Confidence Interval of the difference.

**Table 5:** Centralizer results according to the assessment of behaviour problems—Scale B.

Subscale B	Group	Testing	X	DX (TF-TI) ± SD	CI95%		t	*p*	d
					Lower	Upper			
Self-Centred/Explosive	EG	TI	18,973	−5.564 ± 4.107	−4.521	−6.607	10.667	0.000	0.521
		TF	13,408						
	CG	TI	20,875	0.047 ± 1.404	0.352	−0.257	0.311	0.757	0.147
		TF	20,922						
Attention problems/overactive	EG	TI	14,455	−3.935 ± 2.845	−3.212	−4.657	10.892	0.000	0.438
		TF	10,520						
	CG	TI	15,570	0.261 ± 2.318	0.765	−0.241	1.035	0.304	0.204
		TF	15,831						
Antisocial/Aggressive	EG	TI	13,258	−3.935 ± 2.079	−3.407	−4.463	14.903	0.000	0.403
		TF	9323						
	CG	TI	14,531	−0.380 ± 2.798	0.22626	−0.988	1.248	0.216	0.213
		TF	14,151						
Social Withdrawal	EG	TI	13,632	−2.500 ± 4.679	−1.311	−3.688	4.207	0.000	0.487
		TF	11,132						
	CG	TI	13,276	0.095 ± 1.402	0.399	−0.209	−0.622	0.535	0.198
		TF	13,371						
Anxiety/Somatic Problems	EG	TI	14,532	−2.532 ± 2.934	−1.786	−3.277	6.794	0.000	0.406
		TF	12,000						
	CG	TI	14,372	0.135 ± 1.530	0.415	−0.248	−0.499	0.619	0.189
		TF	14,523						
Total score for behaviour problems	EG	TI	74,844	−18.267 ± 10.288	−15.854	−21.080	14.133	0.000	0.499
		TF	56,372						
	CG	TI TF	78,620 78.323	0.107 ± 4.713	1.130	−0.915	−0.208	0.835	0.261

X—arithmetic mean; DX—the difference between testers; SD—standard deviation; CI95%—95% Confidence Interval of the difference.

## References

[1] OECD (2015). Skills for Social Progress: The Power of Social and Emotional Skills, OECD Skills Studies. https://www.oecd.org/education/skills-for-social-progress-9789264226159-en.htm.

[2] Alexa S., Baciu E.L. (2021). School Dropout and Early School Leaving in Romania: Tendencies and Risk Factors. Rev. Rom. Pentru Educ. Multidimens..

[3] Opre A., Buzgar R. (2012). The efficacy of SELF KIT program in developing socioemotional competencies of kindergarten children. Procedia-Soc. Behav. Sci..

[4] Vancraeyveldt C., Verschueren K., Wouters S., Van Craeyevelt S., Van den Noortgate W., Colpin H. (2015). Improving teacher-child relationship quality and teacher-rated behavioral adjustment amongst externalizing preschoolers: Effects of a two-component intervention. J. Abnorm. Child Psychol..

[5] Moraru A., Stoica M., Tomuletiu E.A., Filpisan M. (2011). Evaluation of a Program for Developing Socio-Emotional Competencies in Preschool Children. Procedia-Soc. Behav. Sci..

[6] Hardy J., Brown J., Skow K. (2015). Early Childhood Behavior Management. https://iris.peabody.vanderbilt.edu/wp-content/uploads/pdf_case_studies/ics_ec_behavior_mgmt.pdf.

[7] Tamm L., Peugh J. (2019). Concordance of teacher-rated and performance-based measures of executive functioning in preschoolers. Child Neuropsychol..

[8] Brodin J., Renblad K. (2015). Early Childhood Educators’ Perspectives of the Swedish National Curriculum for Preschool and Quality Work. Early Childhood Educ. J..

[9] International Standard Classification of Education (ISCED). https://ec.europa.eu/eurostat/statistics-explained/index.php?title=International_Standard_Classification_of_Education_(ISCED)#Implementation_of_ISCED_2011_.28levels_of_education.29.

[10] Anexa la Ordinul Ministrului Educației Naționale nr. 694/2.08.2019. https://www.edu.ro/sites/default/files/Curriculum%20ET_2019_aug.pdf.

[11] Diener M.L., Brehl B.C.W., Black T. (2016). Socioemotional Correlates of Creative Potential in Preschool Age Children: Thinking Beyond Student Academic Assessments. Creat. Res. J..

[12] McClelland M.M., Tominey S.L., Schmitt S.A., Duncan R. (2017). SEL Interventions in Early Childhood. Future Child..

[13] Pianta R.C., Barnett W.S., Burchinal M., Thornburg K.R. (2009). The effects of pre-school education: What we know, how public policy is or is not aligned with the evidence base, and what we need to know. Psychol. Sci. Public Interest.

[14] Raport Privind Starea Invatamantului Preuniversitar din Romania 2019–2020 (Report on the State of Pre-University Education in Romania 2019–2020). https://www.edu.ro/sites/default/files/_fi%C8%99iere/Minister/2020/Transparenta/Stare%20invatamant/Stare%20preuniversitar_rev_5.07.2021.pdf.

[15] Villardón-Gallego L., García-Carrión R., Yáñez-Marquina L., Estévez A. (2018). Impact of the Interactive Learning Environments in Children’s Prosocial Behavior. Sustainability.

[16] Ştefan C.A., Bălaj A., Porumb M., Albu M., Miclea M. (2009). Preschool screening for social and emotional competencies–development and psychometric properties. Cogn. Brain Behav..

[17] Flook L., Goldberg S.B., Pinger L., Davidson R.J. (2015). Promoting prosocial behavior and self-regulatory skills in preschool children through a mindfulness-based Kindness Curriculum. Dev Psychol..

[18] Meyers A.B., Hickey A. (2014). Multilevel Prospective Dynamics in School-Based Social and Emotional Learning Programs. J. Cogn. Educ. Psychol..

[19] Reyna C., Brussino C. (2009). Psychometric properties of the Scale of Preschool and Kindergarten Behavior in a sample of Argentine children from ages 3 to 7. Psykhe.

[20] Lonigan C.J., Spiegel J.A., Goodrich J.M., Morris B.M., Osborne C.M., Lerner M.D., Phillips B.M. (2017). Does preschool self-regulation predict later behavior problems in general or specific problem behaviors?. J. Abnorm. Child Psychol..

[21] Major S.O., Seabra-Santos M.J., Martin R.P. (2015). Are we talking about the same child? Parent-teacher ratings of preschoolers’ social-emotional behaviors. Psychol. Sch..

[22] Neaman A., Otto S., Vinokur E. (2018). Toward an Integrated Approach to Environmental and Prosocial Education. Sustainability.

[23] Ştefan C.A., Miclea M. (2013). Effects of a multifocused prevention program on preschool children’s competencies and behavior problems. Psychol. Sch..

[24] Taylor R.D., Oberle E., Durlak J.A., Weissberg R.P. (2017). Promoting Positive Youth Development through School-Based Social and Emotional Learning Interventions: A Meta-Analysis of Follow-Up Effects. Child Dev..

[25] Kemple K.M., Lee I., Ellis S.M. (2019). The Impact of a Primary Prevention Program on Preschool Children’s Social–Emotional Competence. Early Child. Educ. J..

[26] Romero-López M., Pichardo M.C., Bembibre-Serrano J., García-Berbén T. (2020). Promoting Social Competence in Preschool with an Executive Functions Program Conducted by Teachers. Sustainability.

[27] Funk S., Ho J. (2018). Promoting young children’s social and emotional health. Young Child..

[28] Humphries M.L., Williams B.V., Tanginia M. (2018). Early Childhood Teachers’ Perspectives on Social-Emotional Competence and Learning in Urban Classrooms. J. Appl. Sch. Psychol..

[29] Zhang X., Sun J. (2011). The reciprocal relations between teachers’ perceptions of children’s behavior problems and teacher-child relationships in the first preschool year. J. Genet. Psychol..

[30] Keenan K., Shaw D. (1997). Developmental and social influences on young girls’ early problem behavior. Psychol. Bull..

[31] Eisenberg N., Fabes R.A., Bernzweig J., Karbon M., Poulin R., Hanish L. (1993). The relations of emotionality and regulation to pre-schoolers’ social skills and sociometric status. Child Dev..

[32] Denham S.A., Way E., Kalb S.C., Warren-Khot H.K., Bassett H.H. (2013). Preschoolers’ social information processing and early school success: The challenging situations task. Br J Dev Psychol..

[33] Win S.Y., Nwe K.H. (2020). An analysis of the impact of play on preschool Children’s social skills development. J. Myanmar Acad. Arts Sci..

[34] O’Conner R., De Feyter J., Carr A., Luo J.L., Romm H. (2017). A Review of the Literature on Social and Emotional Learning for Students Ages 3–8: Characteristics of Effective Social and Emotional Learning Programs (Part 1 of 4).

[35] Wyatt J.B., Bloemke G.A. (2013). Social and Emotional Learning in a Freshman Seminar. High. Educ. Stud..

[36] Blewitt C. (2021). Complexity and change: Contemporary research in early childhood. Australas. J. Early Child..

[37] Denham S.A., Weissberg R.P., Chesebrough E., King P., Gullota T.P., Bloom M. (2004). Social-emotional learning in early childhood: What we know and where to go from here. A Blueprint for the Promotion of Prosocial Behavior in Early Childhood.

[38] Blewitt C., Morris H., O’Connor A., Ifanti A., Greenwood D., Skouteris H. (2021). Social and emotional learning in early childhood education and care: A public health perspective. Aust. N. Z. J. Public Health.

[39] Korpershoek H., Harms T., de Boer H., van Kuijk M., Doolaard S. (2016). A Meta-Analysis of the Effects of Classroom Management Strategies and Classroom Management Programs on Students’ Academic, Behavioral, Emotional, and Motivational Outcomes. Rev. Educ. Res..

[40] Shutts K., Kenward B., Falk H., Ivegran A., Fawcett C. (2017). Early preschool environments and gender: Effects of gender pedagogy in Sweden. J. Exp. Child Psychol..

[41] Graziano P.A., Hart K. (2016). Beyond behavior modification: Benefits of social-emotional/self-regulation training for preschoolers with behavior problems. J. Sch. Psychol..

[42] Blewitt C., Fuller-Tyszkiewicz M., Nolan A., Bergmeier H., Vicary D., Huang T., McCabe P., McKay T., Skouteris H. (2018). Social and Emotional Learning Associated with Universal Curriculum-Based Interventions in Early Childhood Education and Care Centers: A Systematic Review and Meta-analysis. JAMA Netw. Open..

[43] Justicia-Arráez A., Pichardo M.C., Romero-López M., Alba G. (2021). Can We Manage Behavioral Problems through the Development of Children’s Social-Emotional Regulated Behavior? Longitudinal Study of a Preschool Program. Int. J. Environ. Res. Public Health.

[44] He M. (2018). Creating Play Atmosphere and Time for Children in Chinese Kindergarten: Difficulties and Reflection. Integr. Psychol. Behav. Sci..

[45] Blewitt C., O’Connor A., Morris H., Mousa A., Bergmeier H., Nolan A., Jackson K., Barrett H., Skouteris H. (2020). Do Curriculum-Based Social and Emotional Learning Programs in Early Childhood Education and Care Strengthen Teacher Outcomes? A Systematic Literature Review. Int. J. Environ. Res. Public Health..

[46] Domitrovich C., Durlak J., Staley K., Weissberg R. (2017). Social-emotional competence: An essential factor for promoting positive adjustment and reducing risk in school children. Child Dev..

[47] Merrell K.W. (2002). Preschool and Kindergarten Behavior Scales.

[48] Major S.O., Seabra-Santos M.J., Martin R.P. (2022). Differentiating Preschoolers with (out) Social-Emotional and Behavioral Problems: Do We Have a Useful Tool?. Assess. Eff. Interv..

[49] Kaya I., Deniz M.E. (2020). The effects of life skills education program on problem behaviors and social skills of 4-year-old preschoolers. Elem. Educ. Online.

[50] Alwaely S.A.A., Yousif N.B.A., Mikhaylov A. (2021). Emotional development in preschoolers and socialization. Early Child Dev. Care.

[51] Tersi M., Matsouka O. (2020). Improving Social Skills through Structured Playfulness Program in Preschool Children. Int. J. Instr..

[52] Langeveld J.H., Gundersen K.K., Svartdal F. (2012). Social competence as a mediating factor in reduction of behavioral problems. Scand. J. Educ. Res..

[53] Seabra-Santos M.J., Gaspar M.F., Major S.O., Patras J., Azevedo A.F., Homem T.C., Pimentel M., Baptista E., Klest S., Vale V. (2018). Promoting Mental Health in Disadvantaged Preschoolers: A Cluster Randomized Controlled Trial of Teacher Training Effects. J. Child Fam. Stud..

[54] Kırkıç K.A. (2021). Investigation of the Problematic Behaviors of Preschool Students Studying in Public and Private Schools. Int. J. Progress. Educ..

[55] Benítez J.L., Fernández M., Justicia F., Fernández E., Justicia A. (2011). Results of the Aprender a Convivir Program for development of social competence and prevention of antisocial behavior in four-year-old children. Sch. Psychol. Int..

[56] Harrington E.M., Trevino S.D., Lopez S., Giuliani N.R. (2020). Emotion regulation in early childhood: Implications for socioemotional and academic components of school readiness. Emotion.

[57] Pickens J. (2009). Socio-emotional Programme Promotes Positive Behaviour in Preschoolers. Child Care Pract..

[58] Pahigiannis K., Glos M. (2020). Peer influences in self-regulation development and interventions in early childhood. Early Child Dev. Care.

[59] Jankauskiene R., Baceviciene M., Pajaujiene S., Badau D. (2019). Are Adolescent Body Image Concerns Associated with Health-Compromising Physical Activity Behaviours?. Int. J. Environ. Res. Public Health.

[60] Zachariou A., Whitebread D. (2022). The relation between early self-regulation and classroom context: The role of adult presence, the task’s source of initiation, and social context. Br. J. Educ. Psychol..

[61] Maguire L.K., Niens U., McCann M., Connolly P. (2016). Emotional development among early school-age children: Gender differences in the role of problem behaviours. Educ. Psychol..

[62] Agbaria Q. (2020). The Relationship between Self Control Skills and Behavioral Problems among Special Education Primary Grades in Israel. Int. J. Early Child. Spec. Educ..

[63] Major S., Santos S.M.J., Martin R.P., Ventura M.F. (2021). Preschoolers’ social skills and behavior problems: A cross-cultural exploratory study of Angolan and Portuguese teachers’ perceptions. Curr. Psychol..

[64] Hollingsworth H.L., Winter M.K. (2013). Teacher beliefs and practices relating to development in preschool: Importance placed on social–emotional behaviours and skills. Early Child Dev. Care.

[65] Kutasi R. (2021). Teaching Romanian for medical students with the help of interactive methods. Bull. Transilv. Univ. Bras..

[66] Incze R. (2018). The role and importance of narrative in the evaluation of aphasia. Rev. Transilv..

[67] Badau D., Camarda A., Serbanoiu S., Virgil T., Bondoc-Ionescu D., Badau A. (2010). Performance management in sports for all. Int. J. Educ. Inf. Technol..

[68] Kaur J., Sharma A. (2022). Establishing Early Foundations to Promote Emotional Competence in Preschool Children. J. Appl. Soc. Sci..

[69] Schell A., Albers L., von Kries R., Hillenbrand C., Hennemann T. (2015). Preventing Behavioral Disorders via Supporting Social and Emotional Competence at Preschool Age. Dtsch Arztebl Int..

[70] Durlak J.A., Weissberg R.P., Dymnicki A.B., Taylor R.D., Schellinger K.B. (2011). The impact of enhancing students’ social and emotional learning: A meta-analysis of school-based universal interventions. Child Dev..

[71] McCabe P.C., Altamura M. (2011). Empirically valid strategies to improve social and emotional competence of preschool children. Psychol. Sch..

[72] Incze R. (2019). Mărci pragmatice în discursul afazic nonfluent. Rev. Transilv..

[73] Denham S.A., Bassett H.H., Thayer S.K., Mincic M.S., Sirotkin Y.S., Zinsser K. (2012). Observing preschoolers’ social-emotional behavior: Structure, foundations, and prediction of early school success. J Genet Psychol..

[74] Blewitt C., Morris H., Jackson K., Barrett H., Bergmeier H., O’Connor A., Mousa A., Nolan A., Skouteris H. (2020). Integrating Health and Educational Perspectives to Promote Preschoolers’ Social and Emotional Learning: Development of a Multi-Faceted Pro-gram Using an Intervention Mapping Approach. Int. J. Environ. Res. Public Health.

[75] Schindler H.S., Khol-optseva J., Oh S.S., Shonkoff J.P., Yoshikawa H., Duncan G.J., Magnuson K.A. (2015). Maximizing the potential of early childhood education to prevent externalizing behavior problems: A meta-analysis. J. Sch. Psychol..

[76] Downer J., Sabol T.J., Hamre B. (2010). Teacher-child interactions in the classroom: Toward a theory of within and cross-domain links to children’s developmental outcomes. Early Educ. Dev..

[77] Pianta R., Howes C., Burchinal M., Bryant D., Clifford R., Early D., Barbarin O. (2005). Features of pre-kindergarten programs, classrooms, and teachers: Do they predict observed classroom quality and child-teacher interactions?. Appl. Dev. Sci..

[78] Blewitt C., O’Connor A., Morris H., Nolan A., Mousa A., Green R., Ifanti A., Jackson K., Skouteris H. (2021). “It’s Embedded in What We Do for Every Child”: A Qualitative Exploration of Early Childhood Educators’ Perspectives on Supporting Children’s Social and Emotional Learning. Int. J. Environ. Res. Public Health.

[79] Ng S.C., Bull R. (2018). Facilitating social emotional learning in kindergarten classrooms: Situational factors and teachers’ strategies. Int. J. Early Child..

